# Elastic, Viscoelastic and Fibril-Reinforced Poroelastic Material Properties of Healthy and Osteoarthritic Human Tibial Cartilage

**DOI:** 10.1007/s10439-019-02213-4

**Published:** 2019-01-28

**Authors:** Mohammadhossein Ebrahimi, Simo Ojanen, Ali Mohammadi, Mikko A. Finnilä, Antti Joukainen, Heikki Kröger, Simo Saarakkala, Rami K. Korhonen, Petri Tanska

**Affiliations:** 1 Department of Applied Physics, University of Eastern Finland, POB 1627, 70211 Kuopio, Finland; 2 Research Unit of Medical Imaging, Physics and Technology, Faculty of Medicine, University of Oulu, Oulu, Finland; 3 Kuopio University Hospital, Kuopio, Finland

**Keywords:** Articular cartilage, Osteoarthritis, OARSI grade, Stress-relaxation, Dynamic testing, Finite element analysis

## Abstract

Articular cartilage constituents (collagen, proteoglycans, fluid) are significantly altered during osteoarthritis (OA). A fibril-reinforced poroelastic (FRPE) material model can separate the contribution of each constituent on the mechanical response of cartilage. Yet, these properties and their OA related alterations are not known for human tibial cartilage. To answer this gap in the knowledge, we characterized the FRPE as well as elastic and viscoelastic properties of healthy and osteoarthritic human tibial cartilage. Tibial osteochondral explants (*n* = 27) harvested from 7 cadavers were mechanically tested in indentation followed by a quantification of elastic, viscoelastic and FRPE properties. Then they were histopathologically OARSI graded for the severity of OA. FRPE modeling revealed that non-fibrillar matrix modulus was higher in the *healthy* group compared to the *early OA* (*p* = 0.003) and *advanced OA* (*p* < 0.001) groups. The initial fibril network modulus was also higher in the *healthy* group compared to the *early OA* (*p* = 0.009) and *advanced OA* (*p* < 0.001) groups. The permeability correlated with the OARSI grade (*p* = 0.002, *r* = 0.56). For the first time, the FRPE properties were characterized for human tibial cartilage. This knowledge is crucial to improve the accuracy of computational knee joint models.

**Electronic supplementary material:**

The online version of this article (10.1007/s10439-019-02213-4) contains supplementary material, which is available to authorized users.

## Introduction

Articular cartilage, covering the endplates of the articulating bones, provides smooth movements in human joints. The main constituents contributing to the mechanical function of cartilage are negatively charged proteoglycans (PGs), collagen fiber network and interstitial fluid.^31^ During instantaneous or cyclic compressive loading, the pressurization of the interstitial fluid together with the collagen fiber network are the main constituents controlling the tissue stiffness.^1^,^40^ On the other hand, under long-term loading, the interstitial fluid flows out of the tissue and the PGs are mainly responsible for the stiffness of the tissue.^30^

Osteoarthritis (OA) is a degenerative joint disease in which all these constituents experience substantial changes resulting in an altered mechanical function of the cartilage tissue. During OA development, especially the superficial zone of cartilage experiences drastic changes, such as fibrillation of the collagen fiber network together with decreased PG and collagen contents and increased interstitial fluid content.^5^,^19^,^20^,^39^ These changes result in a decrease in the equilibrium and dynamic moduli as well as an increase in tissue permeability, which will reduce the load-bearing capacity of cartilage.

Mechanical alterations in the cartilage properties at different stages of OA have received a great research focus. However, the majority of the studies are based on animal models,^8^,^18^,^26^ and only a limited amount of investigations have been conducted with human tissue. In addition, most of these studies have only considered the evolution of elastic properties without considering the changes in the time-dependent and constituent-specific properties.

Several material models have been developed to characterize the mechanical behavior of articular cartilage. Among them, e.g., the fibril-reinforced poroelastic (FRPE) material model is capable of separating the contribution of the main constituents of the tissue (i.e., PG, collagen and fluid) and their effect on the mechanical response of the tissue.^20^ However, the current knowledge on these constituent-specific mechanical properties and how they alter during OA progression, especially in human tissue, is lacking. Some data exists for human hip joint^27^ and patellar cartilage,^14^ but these properties are not known for femoral or tibial cartilage in the knee joint. Although, simple mechanical properties of human tibial cartilage have been reported previously in literature (i.e., elastic and viscoelastic^6^,^19^ as well as hyperelastic^37^), the constituent-specific material properties of human tibial cartilage are not known. Thus, detailed information about the constituent-specific mechanical properties is highly important for the accurate and detailed modeling of knee joint mechanics. Changes in these properties, e.g., during OA progression, might substantially alter the model outcome. In addition, the constituent-specific material properties can be useful for cartilage tissue engineering approaches.

The primary purpose of this study was to characterize the elastic and viscoelastic properties as well as the constituent-specific fibril-reinforced poroelastic properties of healthy and osteoarthritic human tibial cartilage. For this aim, stress-relaxation and sinusoidal mechanical tests were conducted in indentation geometry to determine the “traditional” elastic and viscoelastic material properties. Furthermore, the same data were used for tissue-level finite-element (FE) analysis in order to determine the constituent-specific material properties. The secondary purpose was to characterize changes in these material properties as a function of OA progression. For this, we conducted histopathological OARSI grading. This study provides quantitative information on human tibial cartilage properties and alterations in these properties occurring during OA progression.

## Materials and Methods

**Figure 1:**
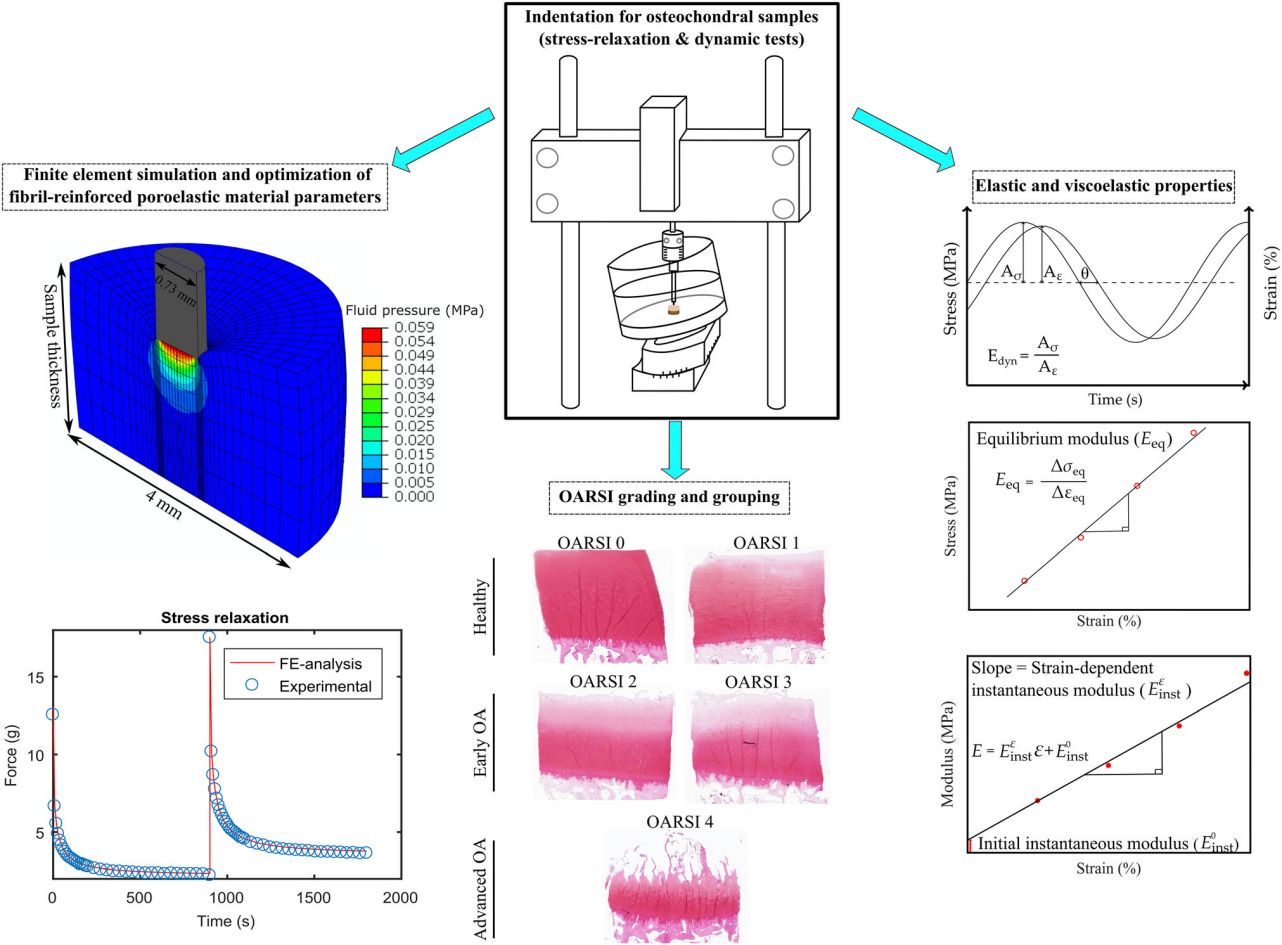
Workflow of the study. Osteochondral samples were prepared from cadavers’ knees, after which the stress-relaxation and dynamic tests were performed for the samples. Elastic and viscoelastic mechanical properties were measured. Subsequently, Safranin-O stained histological sections were prepared from the samples, from which OARSI grades were determined. Sample-specific finite-element models were constructed to extract fibril-reinforced poroelastic material properties through optimization.

Overview of the workflow of this study is presented in Fig. 1.

### Sample Preparation

Cylindrical osteochondral samples (*d* = 4 mm, *n* = 27) were extracted from the tibia of seven human cadavers (age 71.4 ± 5.2 years, range 68–79 years, sex: 6 males and 1 female) at Kuopio University Hospital, Kuopio, Finland. The process and use of the human tissue were approved by the National Authority for Medicolegal Affairs and the ethical committee of North Savo Hospital District (Ethical Permission Number 134/13.02.00/2015). The samples were kept moist and immersed in phosphate buffered saline (PBS, pH 7.4) with enzymatic inhibitors^23^ upon the harvesting process. Thereafter, the samples were stored at − 23 °C in separate sealed containers.

### Mechanical Indentation Testing

Prior to biomechanical indentation tests, the samples were thawed at the room temperature for 15 min. The sample thickness from the cartilage surface to the calcified cartilage, perpendicular to the surface was measured using an optical microscope (× 1.6, Zeiss, STEMI, SV8, Germany) from 4 different quarters around the sample plug. The mean value was then calculated for the sample thickness. To prepare the samples for indentation testing, the bone end was flattened using sandpaper (Mirox P80, Mirka Oy, Uusikaarlepyy, Finland) and then glued to the bottom surface of a custom-made transparent acrylic chamber, filled with the aforementioned PBS medium.^23^

The indentation device used for the biomechanical testing was a custom-made high-precision linear servo-motorized material testing device (Newport PM500-C Precision Motion Controller, Newport PM1A1798 Actuator, Irvine, CA, USA) equipped with a 250 g load cell (Honeywell Model 31/AL311BL, Columbus, OH, USA) and a custom-made cylindrical plane-ended indenter (diameter = 0.73 mm).

To ensure proper sample-indenter contact for consistent and repeatable measurements, a pre-stress of 12.5 kPa was applied to all samples, similarly with previous studies.^21^,^26^ Due to the indenter with a very small diameter, this pre-stress corresponded roughly to 0.005 N force (~ 0.5 g), causing only small deformation to the samples (from 10 to 100 *μ*m depending on the sample thickness and stiffness). This pre-stress caused only slightly more deformation (< 3%) to the degenerated samples compared to the healthy samples. Afterwards, we allowed the sample to equilibrate for ~ 15 min.^7^,^15^ The zero-strain level (reference strain) was set after applying the pre-stress. Then a 4-step stress-relaxation protocol was applied to the sample. Each step consisted of 5% strain (of the remaining cartilage thickness) followed by 15 min of relaxation.^26^ In preliminary tests, this was enough to reach equilibrium. In the compression phase of each stress-relaxation step, a strain rate of 100%/s was applied.^26^,^29^ At the end of the final relaxation step, the dynamic sinusoidal test was carried out using 2% of the remaining thickness with frequencies of 0.005, 0.05, 0.1, 0.25, 0.5, 0.625, 0.833 and 1 Hz. Four cycles at each frequency were conducted.

### Elastic and Viscoelastic Properties

The equilibrium modulus of cartilage was calculated from the slope of the linear least-squares fit to the equilibrium stress–strain points (Fig. 1). Based on a previous study which reported an optically measured Poisson’s ratio for bovine tibial cartilage,^18^ the Poisson’s ratio at equilibrium was set to 0.3. To the best of our knowledge, there are no studies that have optically determined the Poisson’s ratio for human tibial cartilage (at equilibrium and in compression). In order to calculate the dynamic moduli at different frequencies, the stress and strain amplitudes were obtained from each cycle and averaged over four consecutive cycles (Fig. 1). The dynamic modulus was calculated by dividing stress and strain. The samples were assumed incompressible (Poisson’s ratio = 0.5) in the dynamic tests.^16^ The measured equilibrium and dynamic moduli were corrected using the Hayes equation^11^ which takes into account the measurement geometry. The phase difference between the applied stress and strain was calculated from the frequency content of dynamic data using Fourier transform. The phase angle was selected at a frequency at which the power amplitude in the spectrum was the highest. The subtraction of the displacement and the force phase angles was considered as the phase difference. The instantaneous modulus of cartilage was calculated from each peak stress point. Thus, we can obtain data points for the instantaneous modulus as a function of the applied strain. Accordingly, the initial and strain-dependent instantaneous moduli were obtained from the constant term and the slope of the linear least-squares fit to this instantaneous modulus vs. applied strain data (Fig. 1). Due to high loading rate, the samples were assumed incompressible and the Poisson’s ratio was set to 0.5.^16^ The obtained initial and strain dependent instantaneous moduli were also corrected using the Hayes equation.^11^

### Finite Element Analysis and Optimization

The FE models were constructed in Abaqus (V6.14, Dassault Systèmes Simulia Corp., Providence, RI). Cartilage tissue was modeled using the FRPE material model, in which articular cartilage is composed of a porous hyperelastic non-fibrillar matrix (the PG matrix), filled with fluid and an elastic fibrillar matrix (the collagen fiber network). The collagen network was modeled with 4 organized collagen fibrils (primary fibrils) and 13 randomly oriented fibrils (secondary fibrils).^26^,^45^ The ratio of the density of the primary fibrils to the density of the secondary fibrils was set to 12.16.^13^,^26^ The stress of the fibrils was set to zero in compression, while in tension the stress strain-behavior was assumed as non-linear (based on our preliminary analyses) according to:

1 $$\sigma_{\text{f}} = \frac{1}{2}E_{\text{f}}^{{{\varepsilon }}} \varepsilon_{\text{f}}^{2} + E_{\text{f}}^{0} \varepsilon_{\text{f}} ,$$

where $\sigma_{\text{f}}$ and $\varepsilon_{\text{f}}$ are stress and strain of the fibril, $E_{\text{f}}^{0}$ is the initial fibril network modulus and $E_{\text{f}}^{{{\varepsilon }}}$ is the strain-dependent fibril network modulus.^25^ The non-fibrillar matrix was modeled using Neo-Hookean hyperelastic material, in which the non-fibrillar matrix stress is:

2 $${\varvec{\upsigma}}_{\text{nf}} = \frac{1}{2}K_{\text{nf}} \left(J-J^{-1}\right){\mathbf{I}} + \frac{{G_{\text{nf}} }}{J}\left( {{\mathbf{FF}}^{\text{T}} - J^{{\frac{2}{3}}} {\mathbf{I}}} \right),$$

where ${\varvec{\upsigma}}_{\text{nf}}$ is the stress tensor of the non-fibrillar matrix, $G_{\text{nf}}$ and $K_{\text{nf}}$ are the shear and bulk moduli of the non-fibrillar matrix, respectively, **F** is the deformation gradient tensor, *J* is the determinant of the **F** and **I** is the unit tensor.^44^ The bulk and shear moduli of the non-fibrillar matrix can be expressed as a function of Young’s modulus ($E_{\text{nf}}$) and Poisson’s ratio ($\nu_{\text{nf}} = 0.42$, based on Refs. 20, 26) of the non-fibrillar matrix:

3 $$K_{\text{nf}} = \frac{{E_{\text{nf}} }}{{3(1 - 2\nu_{\text{nf}} )}} ,$$

4 $$G_{\text{nf}} = \frac{{E_{\text{nf}} }}{{2(1 + \nu_{\text{nf}} )}} ,$$

Darcy’s law^12^ was employed to describe the fluid flow inside the porous matrix as follows:

5 $$q = - k\nabla p,$$

where *q* is the rate of the fluid flow, $k$ is the (hydraulic) permeability of the material and $\nabla p$ is the (fluid) pressure gradient. Darcy’s law is valid with laminar and low velocity flows, which is true in most biological tissues.^9^ The deformation in the porous material causes a change in the void ratio (the proportion of the fluid volume to the solid volume) and, consequently, the permeability changes, which is described as^42^:

6 $$k = k_{0} \left( {\frac{1 + e}{{1 + e_{0} }}} \right)^{M} ,$$

where $k$ and $k_{0}$ are the current and initial values for the permeability, and $e$ and $e_{0}$ are the current and initial values for the void ratio, respectively. The value for the initial void ratio was set to 3 based on Ref. 22 (corresponding the fluid fraction of 0.75). *M* is a constant describing the void-ratio (or deformation) -dependency of permeability.^9^,^42^,^45^

Sample-specific axisymmetric models were built for FE analysis. The samples were meshed by linear axisymmetric pore pressure continuum elements, counting from 225 to 600 depending on the sample thickness (element type CAX4P). Mesh convergence was ensured. The structure and composition (collagen fiber network orientation, PG and collagen content as well as fluid fraction/void ratio of each sample were assumed as homogenous in order to obtain mechanical material properties independent from the composition and structure of the tissue, similarly as was done before.^20^,^27^ The contact between the bottom of the indenter and the cartilage surface was modeled as a displacement boundary condition for computational efficacy. The contact between the lateral edge of the indenter (an analytical rigid surface) and the cartilage surface was modeled using a frictionless hard contact (both in the normal and tangential direction; during contact the separation of surfaces was allowed only in tangential directions) to prevent folding of the cartilage mesh. Free draining was allowed from non-contacting surfaces (pore pressure = 0), while the cartilage-indenter contact was impermeable. The axisymmetric boundary condition was applied on the symmetry axis of the sample, i.e., lateral displacement of the nodes at the symmetry axis was fixed and fluid was not allowed to flow through the symmetry axis.^26^ Axial and lateral displacements of the bottom nodes of cartilage were fixed as subchondral bone was considered as rigid with respect to cartilage and to simulate subchondral bone-cartilage attachment. No fluid flow was allowed through the bottom surface. The models were solved using a soils consolidation analysis procedure in Abaqus.

The material parameters of the FRPE model ($E_{\text{f}}^{0} , E_{\text{f}}^{\varepsilon } , E_{\text{nf}} , k_{0} , M$) were obtained by optimizing the force-time response of the second and third steps of the model to the corresponding ones in the experimental stress-relaxation test. This means that the first step was considered as a “pre-step”,^26^ i.e., it further ensured proper contact between the tissue and indenter for the model optimization. In addition, if more than two steps are used in an optimization procedure, the high nonlinearities resulting from inherent inhomogeneities of cartilage may become dominant, thus leading to poor optimization performance. The optimization of constituent-specific material properties was conducted using the Nelder-Mead simplex algorithm (*fminsearch*) implemented in Matlab v7.10.0 (The MathWorks, Inc., Natick, MA).^24^ The objective function for the optimization routine was selected as a normalized mean squared error between the simulated and experimental data, which was modified with weighting of error resulting from peaks as follow (the value for the weighting factor *w* = 1 was selected based on preliminary simulations):

$${{\delta }}\bar{F} = \frac{1}{n}\mathop \sum \limits_{i = 1}^{n} \left( {\frac{{F_{i}^{\text{sim}} - F_{i}^{ \exp } }}{{F_{i}^{ \exp } }}} \right)^{2} + w\,\frac{1}{m}\mathop \sum \limits_{j = 1}^{m} \left( {\frac{{F_{{j,{\text{p}}}}^{\text{sim}} - F_{{j,{\text{p}}}}^{ \exp } }}{{F_{{j,{\text{p}}}}^{ \exp } }}} \right)^{2} ,$$

where $F_{i}^{\text{sim}}$ and $F_{i}^{ \exp }$ are simulated and experimental force values, $F_{{j,{\text{p}}}}^{\text{sim}}$ and $F_{{j,{\text{p}}}}^{ \exp }$ are peak force values obtained from the simulation and experiment, and $n$ and $m$ correspond to the total number of data points and number of peak data points ($m = 2$), respectively.

### OA Grading and Grouping of the Samples

Safranin-O stained histological sections were prepared after the experiments. Safranin-O is a stain that binds stoichiometrically to the PGs of cartilage.^17^ The OA severity of the samples was defined using the Osteoarthritis Research Society International (OARSI) histopathological grading system.^36^ Three experts evaluated the samples independently and assigned OARSI grades in consensus to each sample. Our samples were selected so that there was no denudation of articular cartilage (i.e., OARSI grades were between 0 and 4.5). Samples were pooled based on the main OARSI grade (i.e., according to integer grade values) to *healthy* with intact surface (OARSI grades 0–1), *early OA* with fibrillation and minor abrasion of most superficial cartilage (OARSI grades 2–3) and *advanced OA* with fissures, cartilage erosion and cartilage matrix loss in the superficial zone (OARSI grade 4).

### Statistical Analysis

Statistical analyses were conducted to the variables (i.e., equilibrium and dynamic moduli, phase difference as well as the FRPE material parameters ($E_{\text{f}}^{0} , E_{\text{f}}^{\varepsilon } , E_{\text{nf}} , k_{0} , M$)) in order to compare the parameters between different OA progression groups (*healthy, early OA* or *advanced OA* groups based on the OARSI grades). Linear mixed model was used in statistical comparisons. This statistical model considers the dependence of the samples harvested from the same cadaver. In the model, subjects in each group were set as a random effect while the group (*healthy, early OA* or *advanced OA*) was set as a fixed variable. Bonferroni adjustment was conducted for multiple comparisons to obtain conservative estimates for significant differences between the groups. In addition, Spearman’s correlation analysis was conducted to evaluate the relationships between the OARSI grades and the elastic, viscoelastic as well as the FRPE material parameters of cartilage. In all analyses, the level of statistical significance was set at *α* = 0.05. Statistical analyses were performed using IBM SPSS Statistics (version 25, IBM Corporation, Armonk, NY, USA).

## Results

**Figure 2:**
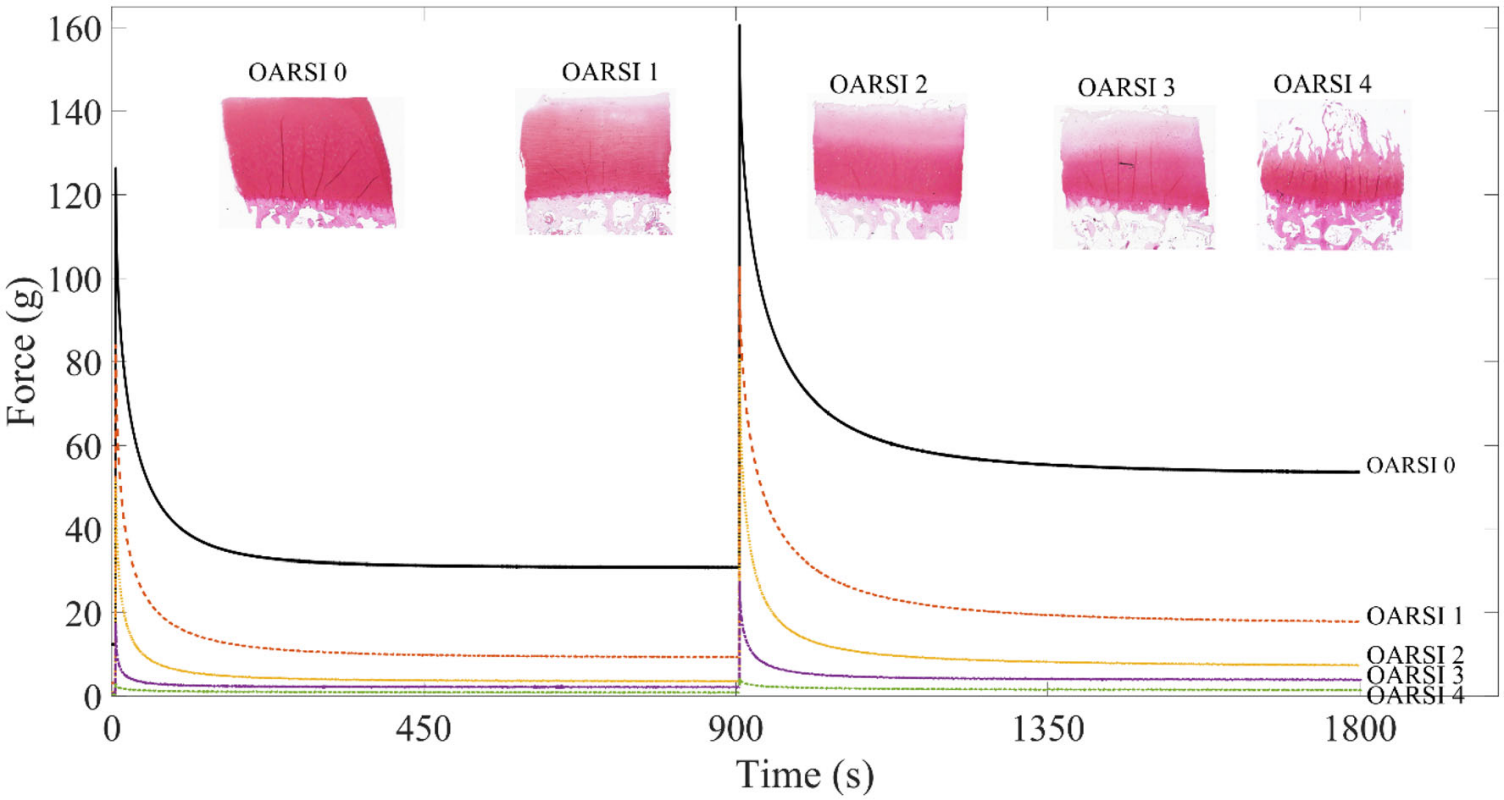
Representative stress-relaxation responses between different OARSI grade samples. Load-bearing capacity of cartilage decreases dramatically with increasing histopathological OA grade as indicated by force.

Figure 2 represents stress-relaxation responses of 5 representative samples, each having different OARSI grade. Degeneration of the samples, as characterized by histology, caused reduced load-bearing capacity of cartilage as seen in the force response of the stress-relaxation curve. All data is presented as mean ± standard deviation.

The FRPE material model could successfully simulate experimental data obtained from indentation tests, measuring *R*^2^ = 0.92 ± 0.12 for the coefficient of determination. The elastic, viscoelastic and FRPE material parameters for each OARSI group (*healthy, early OA* or *advanced OA*) are shown in Table 1. These material parameters are also presented for each main OARSI grade in the supplementary material. See Supplementary material, table S1.

**Table 1:** Obtained FRPE, elastic and viscoelastic (mean ± standard deviation) material parameters for the healthy and OA groups.

Parameter	Healthy: (OARSI 0–1)	Early OA: (OARSI 2–3)	Advanced OA: (OARSI 4)
Number of subjects	*N* = 4	*N* = 5	*N* = 7
Number of samples	*n* = 5	*n* = 7	*n* = 15
$E_{\text{f}}^{0}$ (MPa)	0.41 ± 0.37	0.07 ± 0.17	0.002 ± 0.07
$E_{\text{f}}^{\varepsilon }$ (MPa)	15.42 ± 12.34	18.29 ± 13.89	7.65 ± 6.00
$E_{\text{nf}}$ (MPa)	0.35 ± 0.28	0.10 ± 0.05	0.05 ± 0.04
$k_{0}$ (10^−15^ m^4^ N^−1^ s^−1^)	1.19 ± 0.33	15.94 ± 47.45	20.88 ± 20.34
$M$	3.36 ± 2.07	4.19 ± 3.78	3.52 ± 4.45
$E_{\text{inst}}^{0}$ (MPa)	6.44 ± 4.85	0.42 ± 1.34	− 0.02 ± 0.76
$E_{\text{inst}}^{\varepsilon }$ (MPa)	56.09 ± 33.22	50.05 ± 28.01	21.68 ± 14.12
$E_{\text{eq}}$ (MPa)	1.19 ± 0.56	0.42 ± 0.25	0.21 ± 0.15
$E_{\text{dyn}}$ at 1 Hz (MPa)	6.87 ± 2.57	3.69 ± 2.07	1.67 ± 1.08
*θ* at 1 Hz (^o^)	6.64 ± 0.54	7.40 ± 1.05	8.62 ± 1.94
Thickness	2.83 ± 0.34	3.14 ± 0.90	2.96 ± 0.83

*n* number of samples, *N* number of cadaver subjects, $E_{\text{f}}^{0}$ initial fibril network modulus, $E_{\text{f}}^{\varepsilon }$ strain-dependent fibril network modulus, $E_{\text{nf}}$ non-fibrillar matrix modulus, $k_{0}$ initial permeability, $M$ permeability strain-dependency coefficient, $E_{\text{inst}}^{0}$ initial instantaneous modulus, $E_{\text{inst}}^{\varepsilon }$ strain-dependent instantaneous modulus, $E_{\text{eq}}$ equilibrium modulus, $E_{\text{dyn}}$ dynamic modulus, *θ* phase difference

**Figure 3:**
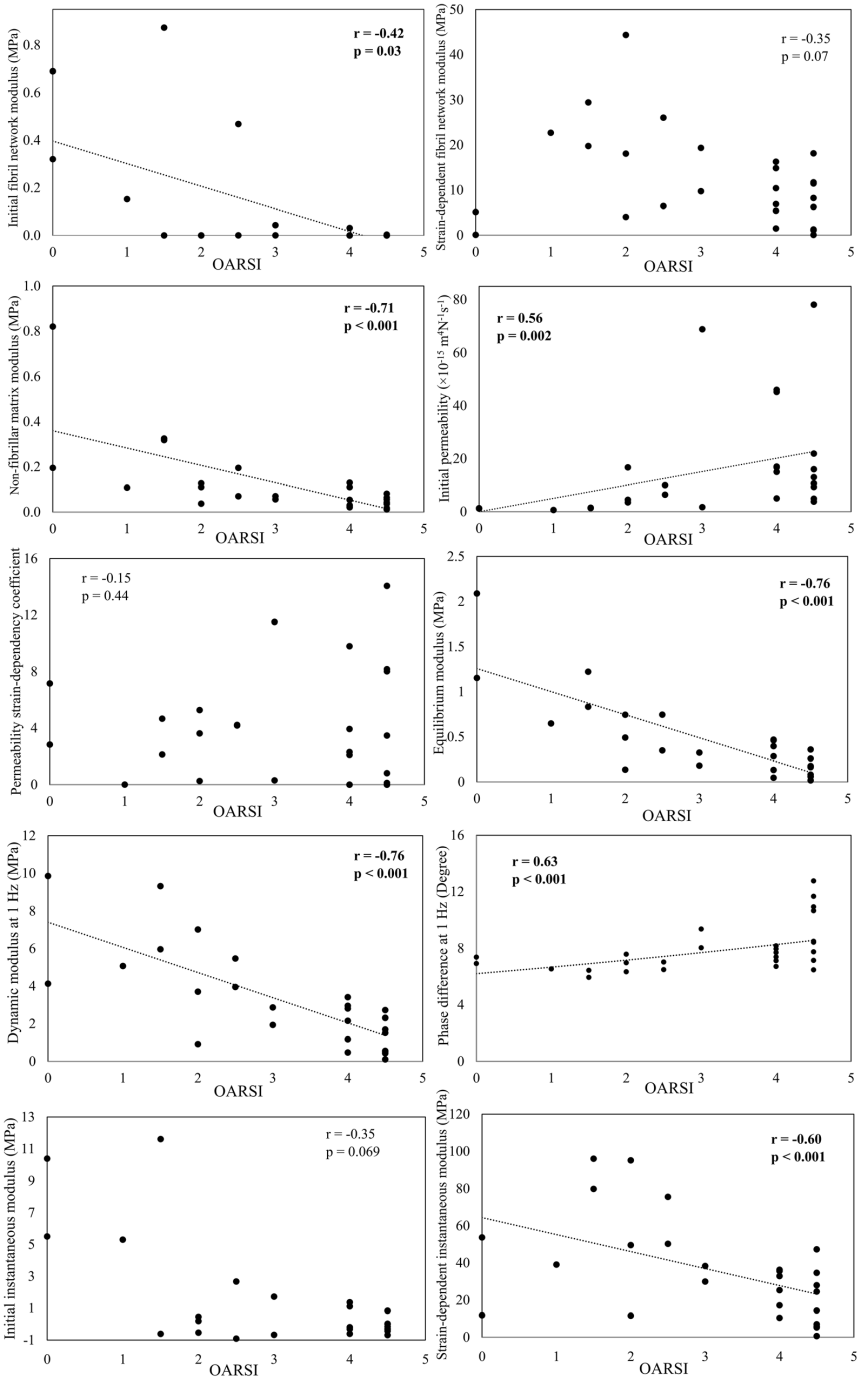
Scatter plots between OARSI grades and elastic and viscoelastic material parameters as well as optimized fibril-reinforced material parameters predicted by the finite element analysis. Statistically significant (Spearman’s) correlations are presented in a bold font.

Significant correlations were observed between the FRPE material parameters and OARSI grades (Fig. 3). The initial fibril network modulus and non-fibrillar matrix modulus demonstrated a significant negative correlation with the OARSI grade (*p* = 0.03, *r* = − 0.42 and *p* < 0.001, *r* = −  0.71, respectively). The initial permeability demonstrated a significant positive correlation with the OARSI grade (*p* = 0.002, *r* = 0.56). The elastic and viscoelastic mechanical parameters were also significantly correlated with the OARSI grade. The equilibrium, dynamic (at 1 Hz) and strain-dependent instantaneous moduli demonstrated a negative correlation with the OARSI grade (*p* < 0.001, *r* = − 0.76; *p* < 0.001, *r* = − 0.76 and *p* < 0.001, *r* = − 0.60, respectively). Similar characteristics for the dynamic moduli were found at all other frequencies (see Supplementary Material Fig. S1). The phase difference showed a significant positive correlation with the OARSI grade (*p* < 0.001, *r* = 0.63). Similar characteristics for the phase difference were found at all frequencies, except at 0.005 Hz (see Supplementary material Fig. S2).

**Figure 4:**
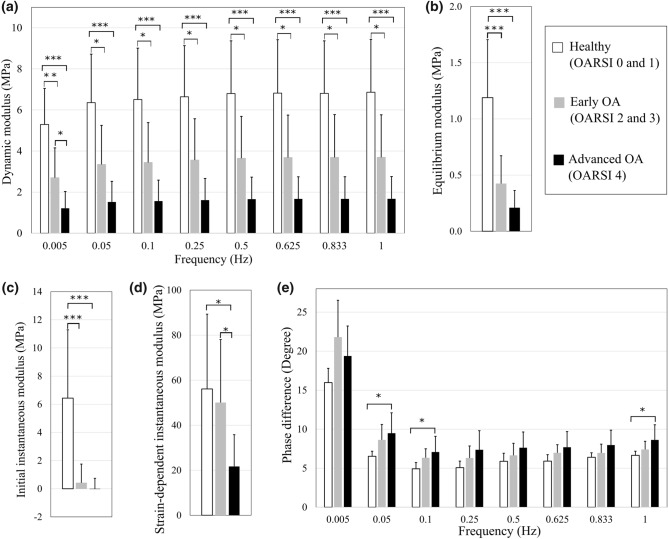
(a) Dynamic, (b) equilibrium, (c) initial instantaneous and (d) strain-dependent instantaneous moduli, and (e) phase difference of the *healthy* (OARSI grades 0 and 1), *early OA* (OARSI grades 2 and 3) and *advanced OA* (OARSI grade 4) groups. *p < 0.05, **p < 0.01 and ***p < 0.001.

Based on the OARSI grading, 5 samples were healthy or very mildly degenerated (OARSI grade 0 or 1), 7 samples were at an early degeneration stage (OARSI grade 2 or 3) and 15 samples exhibited signs of advanced degeneration (OARSI grade 4). The linear mixed model analysis revealed that, at the frequency of 0.005 Hz, the dynamic modulus was significantly smaller in both the *early OA* (*p* = 0.003, 2.70 ± 1.45 MPa) and *advanced OA* (*p* < 0.001, 1.21 ± 0.82 MPa) groups compared to the *healthy* group (5.30 ± 1.75 MPa, Fig. 4a). In addition, the dynamic modulus at this frequency was significantly smaller in the *advanced OA* group compared to the *early OA* group (*p* = 0.036). At the rest of the frequencies (from 0.05 to 1 Hz), the dynamic modulus was significantly greater in the *healthy* group compared to the *early* OA (*p* < 0.05) and *advanced OA* (*p* < 0.05) groups. However, no differences were found between the *early OA* and *advanced OA* groups. Similarly, the equilibrium modulus was significantly greater in the *healthy* (1.19 ± 0.56 MPa) group compared to the *early OA* (*p* < 0.001, 0.42 ± 0.25 MPa) and *advanced OA* groups (*p* < 0.001, 0.21 ± 0.15 MPa, Fig. 4b). Likewise, the initial instantaneous modulus was significantly greater in the *healthy* group (6.44 ± 4.85 MPa) compared to the *early OA* (*p *< 0.001, 0.42 ± 1.34 MPa) and *advanced OA* (*p *< 0.001, − 0.02 ± 0.76 MPa) groups (Fig. 4c). However, the strain-dependent instantaneous modulus was significantly smaller in the *advanced OA* (21.68 ± 14.12 MPa) group than in the *healthy* (*p *= 0.029, 56.09 ± 33.22 MPa) and *early OA* (*p *= 0.041, 50.05 ± 28.01 MPa) groups (Fig. 4d). The phase difference was significantly greater only in the *advanced OA* group compared to the *healthy* group (*p* < 0.05, at the frequencies of 0.05, 0.1 and 1 Hz) (Fig. 4e). No significant differences were found between other groups (*early OA* vs. *advanced OA* or *healthy* vs. *early OA*) or at other frequencies.

**Figure 5:**
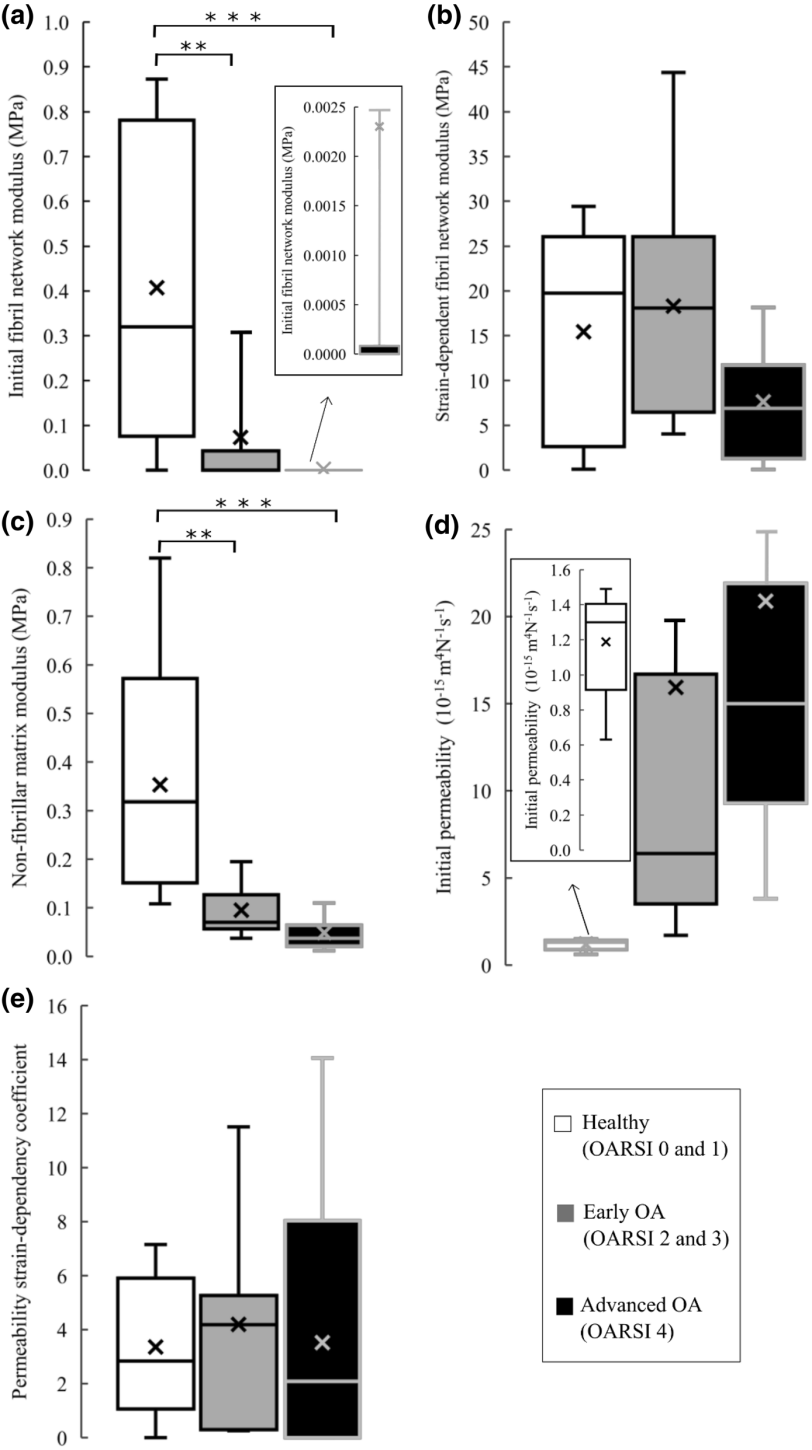
Box plots of the optimized fibril-reinforced poroelastic material parameters for each group; (a) the initial fibril network modulus, (b) strain-dependent fibril network modulus, (c) non-fibrillar matrix modulus, (d) initial permeability, (e) permeability strain-dependency coefficient (Cross = mean value; horizontal line = median value). **p < 0.01 and ***p < 0.001.

The linear mixed model analysis also showed a significantly greater initial fibril network modulus in the *healthy* group compared to the *early OA* (*p* = 0.009) and *advanced OA* (*p* < 0.001) groups (Fig. 5a). Similar to the equilibrium modulus, the non-fibrillar matrix modulus was significantly greater in the *healthy* group compared to the *early OA* (*p* = 0.003) and *advanced OA* groups (*p* < 0.001, Fig. 5c). No significant differences were found between the groups in the strain dependent fibril network modulus, initial permeability or permeability strain-dependency coefficient (Figs. 5b, 5d and 5e).

## Discussion

In the present study, healthy and osteoarthritic human tibial cartilage were characterized by combining indentation testing, tissue-level FE modeling and histological OARSI grading for determining the severity of OA in the samples. Cartilage was modeled by applying the FRPE material model, which enables the evaluation of the contribution of the cartilage constituents (PGs, collagen, fluid) on the mechanical response of the tissue. To the best of our knowledge, we reported the model-derived fibril-reinforced material properties of human tibial cartilage for the first time. Moreover, we established relationships between the elastic, viscoelastic and constituent-specific poroelastic material properties and the progressive state of OA.

OARSI grade has been earlier related to the biomechanical properties of the tissue.^43^ In this study, we showed that the *healthy* group had significantly higher equilibrium and non-fibrillar matrix modulus compared to the *early OA* and *advanced OA* groups, which is often considered as an indicator of higher PG content and fixed charged density within the tissue.^13^,^15^ This is consistent with previously reported correlations between the PG content and the non-fibrillar matrix modulus of cartilage.^13^ This was also seen in our sample set when visually evaluating the Safranin-O stained histological sections. Consistent with our study, previous studies have shown that the equilibrium (or aggregate) modulus is reduced as OA progresses.^19^,^43^

The dynamic modulus was significantly greater in the *healthy* group compared to the *early* *OA* groups and *advanced OA* groups, independent from the frequency of the cyclic loading. This is also consistent with the literature.^43^ Collagen fibers contribute to the dynamic and instantaneous response of cartilage.^40^ Therefore, greater initial fibril network modulus found in the *healthy* group compared to our *OA* groups might explain the greater dynamic modulus in the *healthy* group. We also observed greater initial and strain-dependent instantaneous moduli in the *healthy* group compared to the *advanced OA* group, further supporting dynamic modulus differences.

The initial instantaneous modulus was negative for some samples due to the linear least-squares fitting. This implies that the initial instantaneous modulus for those samples is physically zero, suggesting that the collagen fibers have lost their pretension at the superficial layer. This was also indicated by the FE model, as the initial fibril network modulus for some samples was close to zero.

Interestingly, at the very low frequency (0.005 Hz), the dynamic modulus was significantly higher in the *early OA* group compared to the *advanced OA* group, while at the other frequencies this difference was not significant. Possible reason could be that at the lowest frequency the load is transferred to the non-fibrillar matrix (and thus to PGs) instead of collagen and fluid, thus, providing the significant difference between the *early OA* and *advanced OA* groups. In other frequencies, however, *p*-values were close to 0.05. Low number of samples and high standard deviation in these two groups may explain why we did not observe significant differences.

Interestingly, the strain-dependent fibril network modulus for the samples with OARSI 0 grade (2.26 ± 3.57 MPa) was smaller than that in the rest of the OARSI groups. The OARSI 0 grade samples had presumably no collagen disorganization compared to the samples with a greater OARSI grade. Thus, when we were compressing to the 15% of strain, we were likely measuring more homogenous structure from the samples in the OARSI 0 grade group, as the most part of the sample till this depth was (presumably) intact. In addition, the greater initial network modulus for the OARSI 0 group samples may imply that the pre-tension of the collagen fibrils is the greatest in this OARSI grade group, thus, the collagen fibrils may have been less crimped. This results in the more linear fibril network modulus and, thus, the strain-dependent fibril network modulus is small for the OARSI 0 grade group. Besides, our bulk biomechanical properties also support these observations, as the strain-dependent instantaneous modulus was smaller for the OARSI 0 group compared to the other groups, while the initial instantaneous modulus was the greatest for the OARSI 0 grade group and gradually decreased as a function of the OARSI grade. Based on these interpretations, the strain-dependent fibril network modulus could be an important parameter during OA progression as it can be related with the structural integrity of collagen fibril network (together with the initial fibril network modulus). Yet, based on our results, the strain-dependent fibril network modulus was not different between the pooled OA groups suggesting that it might not be sensitive to OA changes. One reason for this could be the fact that the collagen fibril network is not assessed in the OARSI grading. In order to clarify this issue, we are currently conducting structural and compositional analyses to investigate the role of this parameter during OA progression. Moreover, we acknowledge that only two samples were graded as OARSI 0, which makes it difficult to draw any firm conclusions.

Considering the viscous properties of *healthy* and *advanced OA* cartilage, the *advanced OA* group had significantly greater phase difference at several frequencies, indicating possible changes in the fluid pressurization capability of cartilage.^34^ This can also be related to the altered collagen network integrity and/or reduced swelling capacity of cartilage due to decreased PG content. The degradation and/or fibrillation of the collagen fiber network has been suggested to contribute to the increased permeability^13^,^27^ and reduced fluid pressurization,^35^ which can also result in increasing the phase difference in cartilage. The constituent-specific material properties also support this finding as the permeability was positively correlated with OARSI grade. Recent animal model studies have suggested that the (initial) permeability of cartilage might be susceptible to changes in early OA.^26^ Nonetheless, its strain-dependent behavior was not related to OA progression, possibly because the PG content in all groups was homogeneously distributed at the depths of applied loading (up to 15% of strain) and did not contribute to the non-linearity of tissue permeability at these strains.^41^ This conclusion was supported by visual inspection of Safranin-O stained sections.

The average equilibrium modulus of human tibial cartilage obtained in this study (0.45 ± 0.45 MPa) is consistent with earlier studies characterizing healthy and osteoarthritic human tibial (0.4 MPa^19^), femoral (0.42 MPa^2^) or patellar (0.54 MPa^33^) cartilage. The constituent-specific fibril-reinforced poroelastic material properties obtained in the current study are mainly smaller than or in the range of those reported for cartilage at other sites. The non-fibrillar matrix, the initial fibril network and the strain-dependent fibril network moduli as well as the initial permeability and the permeability strain-dependency coefficient were consistent with the reported values for human hip joint (using healthy and OA samples from femoral neck; Mankin score 5.92 ± 2.87 (mean ± SD) and with values ranging from 2 to 11)^27^ and patellar cartilage^14^,^32^ (using non-osteoarthritic samples). For instance, the mean value of the initial fibril network modulus in this study (0.10 ± 0.22 MPa) was smaller than those reported for human hip joint^27^ (0.59 ± 0.48 MPa) and patellar cartilage^14^ (0.23 ± 0.22 MPa). Similarly, the mean non-fibrillar matrix modulus obtained in this study (0.12 ± 0.16) was smaller than those obtained for human hip joint (0.23 ± 0.22)^27^ and patellar cartilage (0.37 ± 0.24).^14^ However, due to the standard deviations, we cannot be sure of these differences, but rather acknowledge that they are in the same range. It should also be noted that the values reported from human hip joint cartilage were obtained from a set of healthy and OA samples, as Mankin score ranged from 2 to 11, which is similar to our set of samples. The values reported from human patellar cartilage were obtained from non-osteoarthritic samples, which might have resulted in obtaining greater values of constituent-specific material properties. Nonetheless, the constituent-specific material properties of human tibial cartilage in this study were smaller than the values reported for bovine^13^ and white rabbit,^26^ likely because of the natural differences between human and animal tissues.

In previous studies, indentation test has shown a good capability to reveal alterations in tissue properties in the superficial zone of cartilage, where the first OA changes are usually observed.^10^,^39^ Histological studies have revealed that most of the collagen network fibrillation occurs in the superficial zone during the progression of OA,^3^ further supporting the use of indentation test. Indentation also enables testing intact osteochondral samples in its native environment, in contrast to other measurement geometries such as unconfined compression which requires cartilage to be cut from the subchondral bone and might cause additional damage to the tissue.

Compressive non-linear behavior of articular cartilage is typically characterized through the strain-dependency of permeability and collagen fiber stiffness.^45^ In this study, we simulated and optimized two steps of stress-relaxation. If the FRPE model would be fit into a single stress-relaxation step, the model would ignore the strain-dependent non-linear properties of articular cartilage.^26^ In contrast, fitting of the FRPE model into two or more stress-relaxation steps has been reported to successfully capture the strain-dependent properties of cartilage, especially the highly nonlinear properties of collagen fibers.^28^

Regarding the limitations of this study, two samples, with no OA related histopathological changes, did not reach the full equilibrium after 15 min of relaxation in their last step, though the relaxation time was chosen based on the literature and preliminary tests.^7^ However, the FRPE material model is not highly sensitive whether the equilibrium is fully reached or not. Furthermore, the amount of healthy, mildly and moderately degenerated samples (OARSI grade 0 (*n* = 2), grade 1 (*n* = 3), grade 2 (*n* = 5) and grade 3 (*n* = 2)) was relatively small compared to advanced degeneration (OARSI grade 4 (n = 15)), which can also be considered as a limitation. For this reason, we decided to pool OARSI grades 0 and 1 as well as grades 2 and 3 into the *healthy* and *early OA* groups, respectively. We performed our statistical comparisons of the material parameters between the pooled groups due to a low number of samples in the OARSI grades 0, 1, 2 and 3. Yet, we acknowledge that the low number of samples even in the pooled groups and the relatively large variations in the model-derived parameters (that arise primarily from the grouping) may have affected the results. These were most likely the main reasons why we were not able to detect significant differences in some of the model-derived material parameters, e.g., in the initial permeability, although it exhibited a moderate positive correlation (*r* = 0.56, *p* = 0.002) with the OARSI grade and was on average 15 and 20 times greater in the *early OA* and *advanced OA* groups compared to the *healthy* group, respectively.

In order to keep the optimization of the constituent-specific material parameters independent of the structure and composition of the tissue, we assumed a homogenous structure for the samples in the finite element analysis. For instance, collagen orientation (together with permeability) is the main contributor to the peak force in a stress-relaxation test. A sample containing a high amount of collagen fibrils oriented parallel to the surface would show a greater force response compared to a sample containing a high amount of collagen fibrils oriented perpendicular to the surface. If the real structure would be considered in the model and optimization, the sample with more parallel oriented fibrils would produce a greater force response even with the same values of the collagen fibril network modulus. For these two samples, softer and stiffer, we might obtain the same material parameters for collagen and the obtained material parameters would not reflect the differences in biomechanics. Moreover, it is not possible to obtain a unique set of depth-wise mechanical properties of cartilage samples using indentation and optimization.

Prediction of OARSI grades based on the presented biomechanical properties (fibril-reinforced poroelastic and/or elastic–viscoelastic material properties) or prediction of the biomechanical properties based on histology can be potential applications of this study in the future. Multivariate regression models may be developed to predict OARSI grades based on predictor variables (i.e., fibril-reinforced poroelastic and bulk biomechanical properties), or vice versa. However, low number of samples might make it unfeasible to draw a firm conclusion and future investigations are needed with a greater number of samples.

Taken together, the present study provides novel information about the constituent-specific fibril-reinforced poroelastic material properties of human tibial cartilage during the progression of OA, defined by histopathological OARSI grades. The results suggest that the degeneration of cartilage is related with the decrease in the non-fibrillar matrix (contribution of PGs) and fibrillar matrix (contribution of collagen) moduli, while the initial permeability simultaneously increases. Furthermore, the increase in the OARSI grade was related with the decrease in the elastic (equilibrium/dynamic/instantaneous modulus) and increase in the viscous (phase difference) properties of cartilage.

In the future, this information will be combined with a detailed analysis of the structure and composition of cartilage in order to quantify the presumably complex structure–function relationships in human tibial cartilage. The depth-wise analysis of the structure and composition are also important for many *in vitro* and *in situ* studies that investigate strains or stresses of cartilage and chondrocytes in different tissue layers.^4^,^38^ The constituent-specific material properties of human tibial cartilage can be useful for cartilage tissue engineering approaches. Furthermore, implementation of the constituent-specific material parameters with tissue composition and structure into computational models of the human knee joint will improve the accuracy of the models in a depth-wise manner.

## Electronic supplementary material

Below is the link to the electronic supplementary material.

Supplementary material 1 (PDF 376 kb)
