# Supplementary material for: Elastic, Viscoelastic and Fibril-Reinforced Poroelastic Material Properties of Healthy and Osteoarthritic Human Tibial Cartilage
Source: Ann Biomed Eng. 2019 Jan 28;47(4):953–66. doi: 10.1007/s10439-019-02213-4 (PMC8494710; doi:10.1007/s10439-019-02213-4)
Supplement: Supplementary file 1 — Supplementary material 1 (PDF 376 kb) [file 10439_2019_2213_MOESM1_ESM.pdf]

Supplementary material for:

Elastic, viscoelastic and fibril-reinforced poroelastic material properties of healthy and osteoarthritic human tibial cartilage

Mohammadhossein Ebrahimi[1,2], Simo Ojanen[1,2], Ali Mohammadi[1], Mikko Finnilä[2], Antti Joukainen[3], Heikki Kröger[3], Simo Saarakkala[2], Rami K. Korhonen[1], Petri Tanska[1]

[1]Department of Applied Physics, University of Eastern Finland, Kuopio, Finland; [2]Research Unit of Medical Imaging, Physics and Technology, Faculty of Medicine, University of Oulu, Oulu, Finland; [3]Kuopio University Hospital, Kuopio, Finland

Results

Elastic, viscoelastic and FRPE material parameters for each OARSI grade are shown in Table S1.

Table S1. Obtained FRPE, elastic and viscoelastic (mean ± standard deviation) material parameters for different OARSI grades

Parameter	**OARSI 0**	**OARSI 1**	**OARSI 2**	**OARSI 3**	**OARSI 4**	**Total**
Number of subjects	N = 2	N = 2	N = 5	N = 2	N = 7	N = 7
Number of samples	n = 2	n = 3	n = 5	n = 2	n = 15	n = 27
E_{f}^{0} (MPa)	0.50 ± 0.26	0.34 ± 0.47	0.09 ± 0.21	0.02 ± 0.03	0.002 ± 0.07	0.10 ± 0.22
$E_{\mathrm{f}}^{\varepsilon}$ (MPa)	2.26 ± 3.57	23.96 ± 4.96	19.79 ± 16.37	14.54 ± 6.79	7.65 ± 6.00	11.85 ± 10.36
E_{nf} (MPa)	0.51 ± 0.44	0.25 ± 0.12	0.11 ± 0.06	0.06 ± 0.01	0.05 ± 0.04	0.12 ± 0.16
k_0 (10^{-15} $m^4N^{-1}s^{-1}$)	1.25 ± 0.07	1.15 ± 0.46	7.07 ± 5.35	35.25 ± 47.45	20.88 ± 20.34	15.85 ± 19.87
M	4.99 ± 3.06	2.26 ± 2.33	3.28 ± 1.91	5.91 ± 7.93	3.52 ± 4.45	3.66 ± 3.82
E_{inst}^{0} (MPa)	7.94 ± 3.45	5.43 ± 6.11	0.37 ± 1.40	0.52 ± 1.69	-0.02 ± 0.76	1.29 ± 3.20
$E_{\mathrm{inst}}^{\varepsilon}$ (MPa)	32.76 ± 29.64	71.64 ± 29.35	56.42 ± 31.47	34.12 ± 5.94	21.68 ± 14.12	35.41 ± 26.09
E_{eq} (MPa)	1.62 ± 0.66	0.90 ± 0.29	0.49 ± 0.26	0.25 ± 0.10	0.21 ± 0.15	0.45 ± 0.45
E_{dyn} at 1 Hz (MPa)	6.99 ± 4.05	6.78 ± 2.24	4.21 ± 2.27	2.40 ± 0.66	1.67 ± 1.08	3.16 ± 2.53
θ at 1 Hz (°)	7.15 ± 0.32	6.30 ± 0.32	6.88 ± 0.49	8.70 ± 0.94	8.62 ± 1.94	7.94 ±1.73
Thickness	2.62 ± 0.28	2.97 ± 0.34	3.20 ± 1.00	3.01 ± 0.90	2.96 ± 0.83	2.98 ± 0.75

n: number of samples, N: number of cadaver subjects, E_{f}^{0}: initial fibril network modulus, $E_{\mathrm{f}}^{\varepsilon}$: strain-dependent fibril network modulus, E_{nf}: non-fibrillar matrix modulus, k_0: initial permeability, M: permeability strain-dependency coefficient, E_{inst}^{0}: initial instantaneous modulus, $E_{\mathrm{inst}}^{\varepsilon}$: strain-dependent instantaneous modulus, E_{eq}: equilibrium modulus, E_{dyn}: dynamic modulus, θ: phase difference.

Spearman's correlation between the OARSI grade and dynamic modulus at different frequencies (Figure S1). As can be seen from the figure, strong correlations are found at all frequencies.

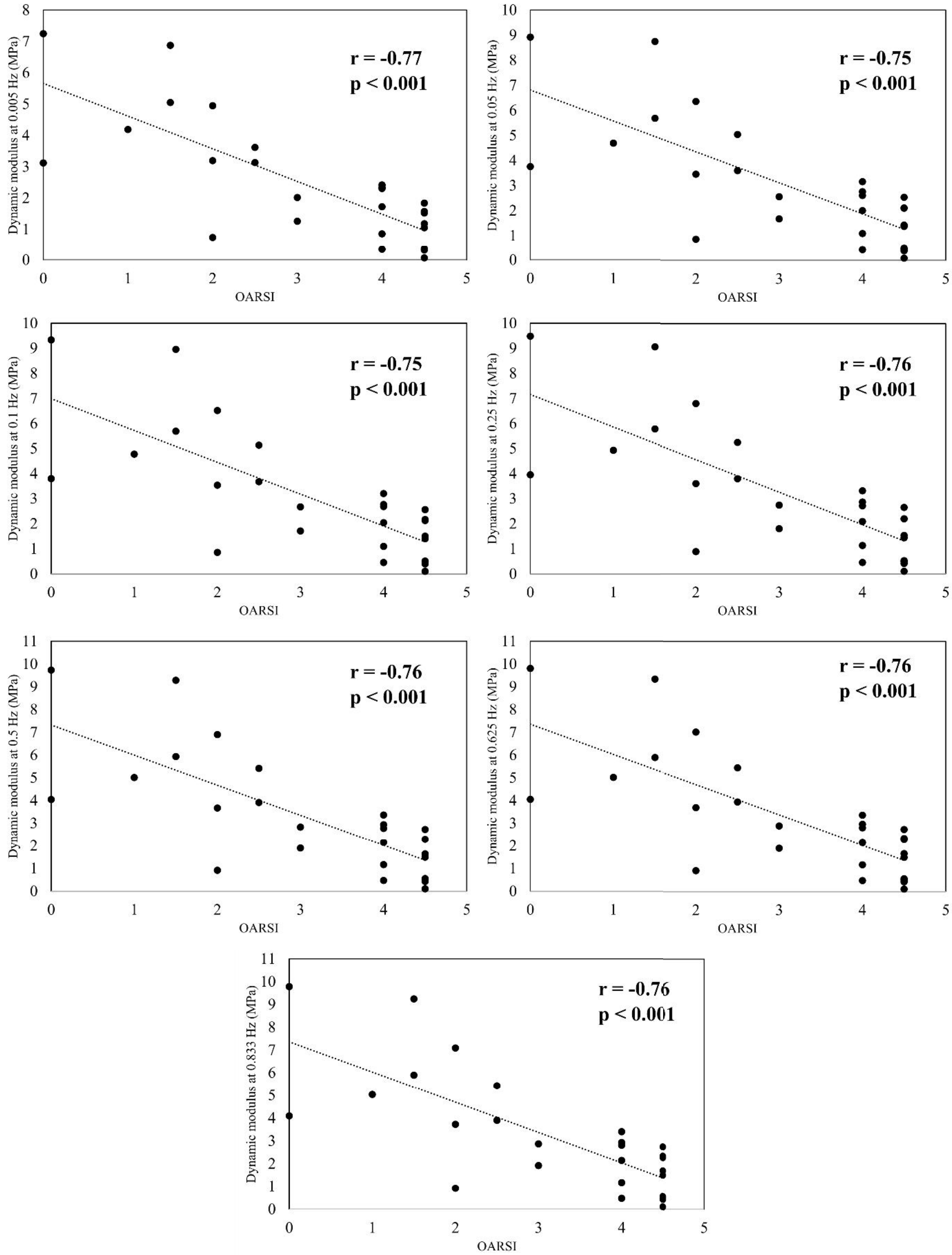

Figure S1: Scatter plots between OARSI grades and dynamic moduli at different frequencies. Statistically significant (Spearman's) correlations are presented in a bold font.

Spearman's correlation between the OARSI grade and phase difference at different frequencies (Figure S2). Moderate positive correlations were found in all frequencies ($p < 0.05$), except at 0.005 Hz ($p = 0.17$).

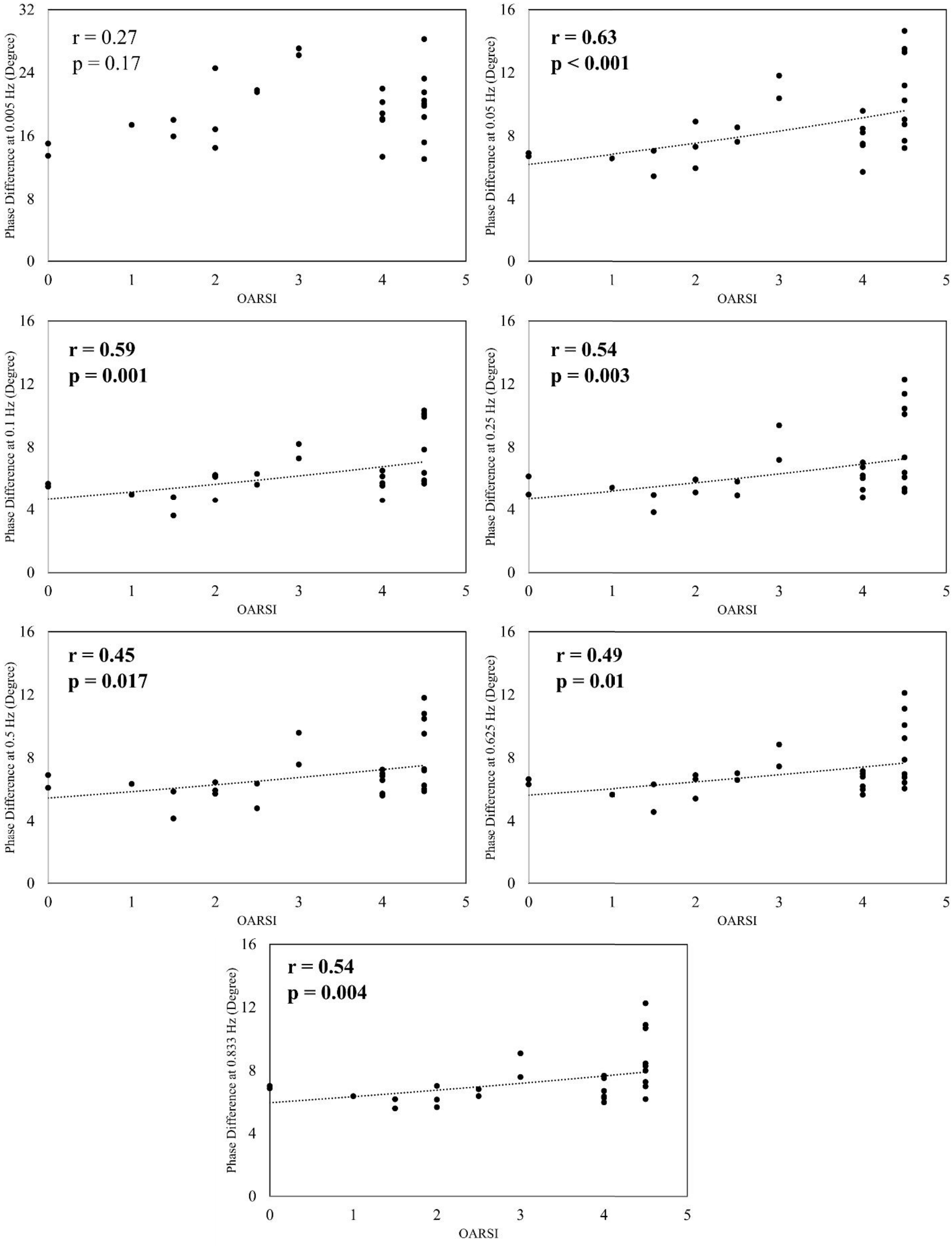

Figure S2: Scatter plots between OARSI grades and phase differences at different frequencies. Statistically significant (Spearman's) correlations are presented in a bold font.
